# Study on hydraulic spray atomizing system as a new resource-efficient dyeing-finishing method for wool fabric

**DOI:** 10.1038/s41598-022-26172-4

**Published:** 2022-12-17

**Authors:** Roos Mulder, Mohammad Neaz Morshed, Sina Seipel, Ulrika Norén, Ellinor Niit, Vincent Nierstrasz

**Affiliations:** 1 Textile Material Technology, Department of Textile Technology, Faculty of Textile, Engineering and Business, The Swedish School of Textiles, University of Borås, 50190 Borås, Sweden; 2 Imogo AB, 216 16 Limhamn, Sweden

**Keywords:** Environmental social sciences, Engineering

## Abstract

This study introduces hydraulic spray (HS) atomizing system as new resource-efficient continuous dyeing-finishing method for wool fabric. Here, wool fabric was dyed and finished by using commercial dyes and finishes through either one-step or two-steps HS method. Results obtained from color strength (K/S), color difference (ΔE_CMC_) and color fastness analysis presented the apprehension of HS method in dyeing of wool fabric with different GSM and dyes. Finishing performance of wool fabric was measured through water contact angle analysis. Analysis shows that, the finishing performance of HS method were substantial to reach water contact angle as high as 145° while maintaining high fastness to wash and abrasion. Between one-step and two-steps HS method, one-step method showed better performance with high resource efficiency compared to two-steps method. Results from statistical analysis shows no statistical significance of fabric weight, type of dyes, and finishes to the performance of new HS method which is crucial for true-scale industrial implementation and scaling up of this process. The findings of this report are of great importance as it presents a greener alternative to the conventional resource-intensive dyeing-finishing methods of wool fabric.

## Introduction

Atomization is essentially the process of converting bulk liquid into small drops. It is a disruption of the consolidating influence of surface tension caused by the action of internal and external forces. Spray atomization is the transformation of a liquid into a spray of fine particles^1^. This process is widely utilized when distributing material over a controlled surface area in various fields due to their high control on the process, low waste generation, and easy operation process. Spraying is the most widely used means of pesticide application for pest control in agriculture and forestry^2^. Recently, hydraulic spraying technology attracted attention to many researchers in functionalization of textiles due to the feasibility, sustainability, and economic benefits^3–5^. Li, Arumugam et al. (2020) reported a fully spray coated organic solar cell fabricated directly on to standard polyester cotton fabric^6^. Samanta and Bordes (2020) proposed a preparation method of conductive textiles by spray coating of water-based graphene dispersions^7^. Sadanandan, Bacon et al. (2020) reported that, spray coating of graphene on textile fabrics is emerging as one of the more promising techniques to overcome the limitations of the irregular and coarse structures of textile fabrics^8^. Spray coating is a potential process for realizing thinner films on textiles. Based on that spray technology can be a recognized alternative to spin coating and non-contact deposition process as opposed to, for example, screen printing. Spray coating also benefits from a wider range of acceptable rheological parameters compared to digital printing, which strictly limits these properties.

The hydraulic spray atomizing system is a continuous process that sprays desired material in the fabric in large through an atomizer^9^. In this system it is no longer necessary to prepare large deposits of chemicals. Besides that, during processing, there are no physical and chemical interaction (as like conventional methods) which protects inherent characteristics of material treated^10^. In addition, the process reduces the discharge of waste as no/less chemicals are needed compared to conventional methods which results in reduction in energy and other resources consumption in subsequent waste management/treatment processes^11,12^.

At present, sustainability aspects in terms of production processes is a serious concern in textile processing industries globally. Out of many challenges in conventional textile processes, resource-insensitivity, waste production and freshwater consumption are the most crucial ones which needs robust and immediate solution. Several advance methods such as liquid ammonia dyeing^13^, supercritical fluid dyeing^14^, crosslinking agents dyeing^15^, foam dyeing^16^, and nanoparticles-based dyeing^17^ and so on^18^ has been introduced for many years but, most of these technologies has not left laboratory environment due to the lack of interest by the relevant business community as these processes either and/or involves drawbacks related to installation cost, low polarity, low capacity batch process, vertical layout, non-homogeneous fluid distribution, chemical accumulation, unreliable operation process, etc.^14,19,20^.

In the pursuit of sustainable upgrade of textile processing into greener production system, there is an urgent need for new advance system that will replace conventional wet production processes while offering prospective of true-scale industrial implementation in terms of feasibility, process engineering, run-length etc.^21^.

Therefore, we propose the hydraulic spray atomizing system as the new method for dyeing and finishing of textiles (wool fabric). Within the best of our knowledge, there are no studies that explored the dyeing-finishing of wool fabric through hydraulic spray atomizing system. Wool fabric was chosen for this study due to their extensive use in clothing, blankets, saddle cloths, insulation, upholstery, technical textiles and so on. Besides, the existing processing wool fabric using acid/reactive dyes is highly resource-intensive which requires a much-needed upgrade into greener alternative.

## Experimental

### Materials

Monosulfonated levelling acid dye (Telon Yellow T-3R) and bi-functional reactive dye (Realan Red EHF) used in this study was purchased from DyStar Pte. Ltd (Singapore). Wetting agent (Rucowet FN), fluorocarbon-free water-repellent finishes ❴Finish 1 (F1): Ruco-Dry ECO DCF, Finish 2 (F2): Ruco-Dry DHE❵ was provided by Rudolf GmbH (Germany). All basic chemicals such as acetic acid (CH_3_COOH), sodium carbonate (Na_2_CO_3_·10H_2_O) and sodium sulphate (Na_2_SO_4_) were of analytic grade and used as received from Sigma Aldrich Ltd. without any further purification. Two different types of wool fabric (Norwegian merino wool); (a) Wool fabric 1 (W1): 469 GSM, washed, milled, sheared, decatized; (b) Wool fabric 2 (W2): 264 GSM, pre-washed, heat set were used.

### Preparation methods of dyeing-finishing of wool fabric (HS and Conventional)

**Figure 1:**
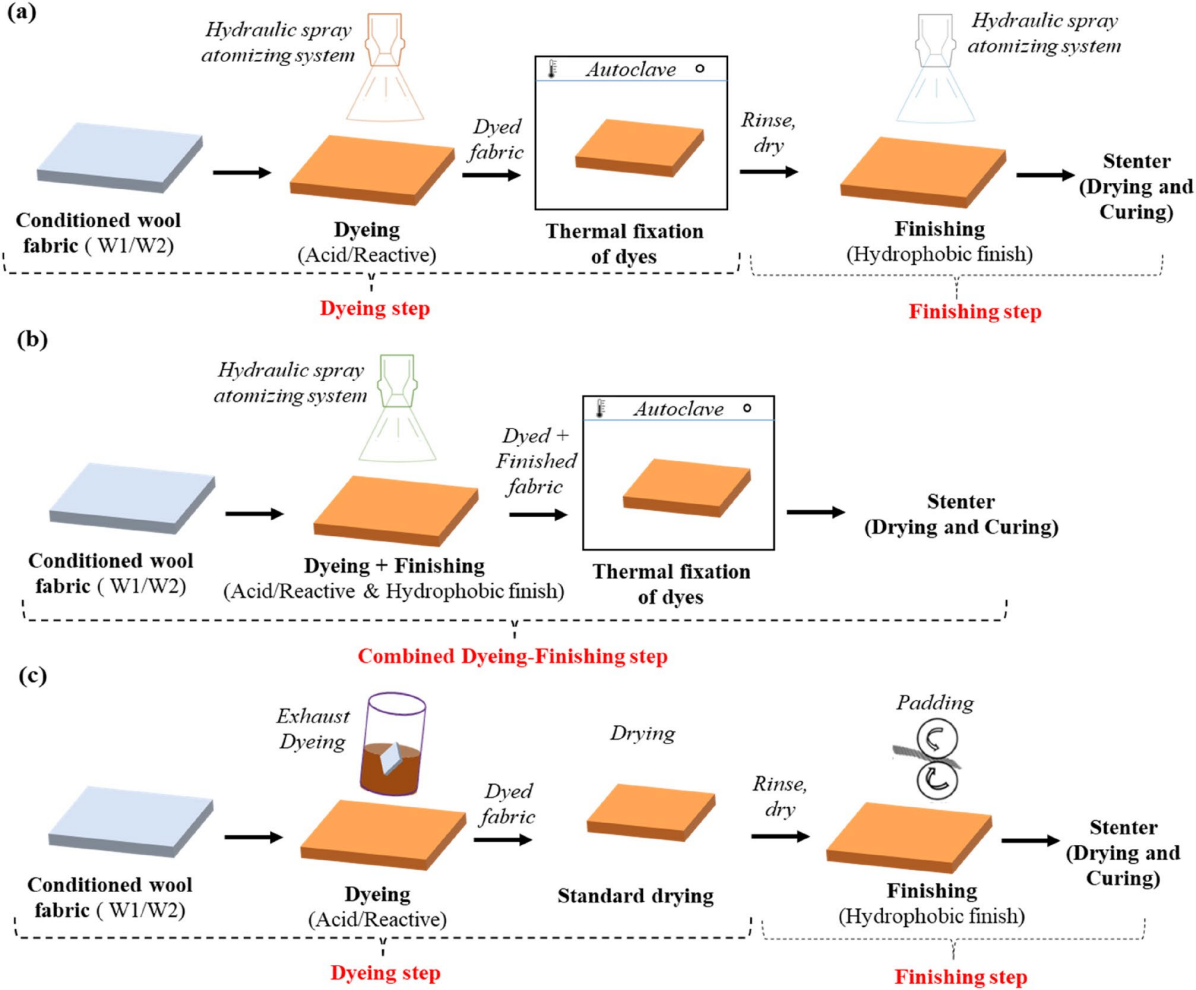
Process steps of (**a**) two-steps dyeing-finishing process of wool fabric through HS method, (**b**) one-step dyeing-finishing process of wool fabric through HS method and (**c**) conventional (two-steps) dyeing-finishing process of wool fabric.

In this study, both one-step and two-steps dyeing-finishing of wool fabric using HS method was studied and compared with conventional processes. MiniMax hydraulic spray atomising system from Imogo AB was used along with laboratory scale FlexDyer. The details of the MiniMax HS atomizing system and the machine parameters can be found in Sect. 1.1 of the *supporting information*. For all studies, samples were conditioned at 20 ± 2 °C temperature and 55 ± 5% relative humidity for 24 h and all parameters were chosen based on respective preliminary studies (see Sect. 1.2 of the *supporting information*). Processes involved in this study are presented schematically in Fig. 1 and described as follows;

- ***Two-steps dyeing-finishing process of wool fabric through HS method:*** In two-steps process, wool fabrics were dyed and finished in separate processes (see Fig. 1a). Spray solution to fabric ratio was 1: 0.4 with 80% pick-up rate followed by standard dye fixation in laboratory autoclave (98 °C for 90 min). Spray solutions were prepared by dissolving either acid dyes (Dye 1: 8.75 g/L, pH ~ 3) or reactive dyes (Dye 2: 20 g/L, pH ~ 4.5) in water. After dyeing, the samples were rinsed and dried at ambient condition before applying the commercial hydrophobic finishes (Finish 1: 80 mL/L, pH ~ 5 and Finish 2: 125 mL/L, pH ~ 4) at 80% pick-up followed by drying (W1: 160 °C for 2 min, W2: 160 °C for 1 min) and curing (W1: 170 °C for 1 min, W2: 170 °C for 0.5 min) in a Mathis lab stenter machine. In this experiment, the samples were however sprayed and finished on both sides.
- ***One-step dyeing-finishing process of wool fabric through HS method:*** Wool fabrics were dyed and finished at the same time at 80% pick-up rate (see Fig. 1b). Dye and finish solution were prepared separately and mixed to set for four spray solution involving two dyes and two commercial hydrophobic finishing agents as: Acid + F1 (pH 3.5), Acid + F2 (pH 3.5), Reactive + F1 (pH 4.5), Reactive + F2 (pH 4.5). The spray was applied on both sides of the fabric before fixation in laboratory autoclave (98 °C). After fixation, the samples were dried and cured in a Mathis lab stenter machine as same as two-steps method.
- ***Conventional dyeing-finishing process of wool fabric:*** Wool fabrics were dyed in an exhaust dyeing machine followed by finishing through a pad-dry-cure method (see Fig. 1c). Typically for dyeing, the liquor ratio was 1: 20. The solution was prepared by dissolving either acid dye (0.35 g/L) or reactive dye (0.8 g/L) and fabrics were dyed following respective standard dyeing curves (Acid dye: pH ~ 3.2, 98 °C, 90 min; Reactive dye: pH ~ 4.5, 98 °C, 60 min). For finishing, the commercial hydrophobic finishes were applied at 60% pick-up rate with a pressure of two bar. Resultant samples were cured in a Mathis lab stenter machine (W1 + F1: 160 °C for 2 min + 170 °C for 1 min, W1 + F2: 140 °C for 3 min, W2 + F1: 160 °C for 1 min + 170 °C for 0.5 min, W2 + F2: 140 °C for 2 min).

In total 24 samples were prepared and comparatively investigated in this study to understand the feasibility of the HS atomizing method for dyeing-finishing of a wool fabric as an advanced resource-efficient process. Summary of all samples and their corresponding descriptions is provided in Table 1.

**Table 1:** Summary of sample name and corresponding descriptions.

Sample name	Descriptions		
	Dye type	Finish type	Dyeing-finishing method
**469 GSM wool fabric (W1)**			
C-W1@AF1	Acid (A)	Ruco-Dry ECO DCF (F1)	Two-steps conventional method
HS1-W1@AF1			One-step HS atomizing method
HS2-W1@AF1			Two-steps HS atomizing method
C-W1@AF2		Ruco-Dry DHE (F2)	Two-steps conventional method
HS1-W1@AF2			One-step HS atomizing method
HS2-W1@AF2			Two-steps HS atomizing method
C-W1@RF1	Reactive (R)	Ruco-Dry ECO DCF (F1)	Two-steps conventional method
HS1-W1@RF1			One-step HS atomizing method
HS2-W1@RF1			Two-steps HS atomizing method
C-W1@RF2		Ruco-Dry DHE (F2)	Two-steps conventional method
HS1-W1@RF2			One-step HS atomizing method
HS2-W1@RF2			Two-steps HS atomizing method
**264 GSM wool fabric (W2)**			
C-W2@AF1	Acid (A)	Ruco-Dry ECO DCF (F1)	Two-steps conventional method
HS1-W2@AF1			One-step HS atomizing method
HS2-W2@AF1			Two-steps HS atomizing method
C-W2@AF2		Ruco-Dry DHE (F2)	Two-steps conventional method
HS1-W2@AF2			One-step HS atomizing method
HS2-W2@AF2			Two-steps HS atomizing method
C-W2@RF1	Reactive (R)	Ruco-Dry ECO DCF (F1)	Two-steps conventional method
HS1-W2@RF1			One-step HS atomizing method
HS2-W2@RF1			Two-steps HS atomizing method
C-W2@RF2		Ruco-Dry DHE (F2)	Two-steps conventional method
HS1-W2@RF2			One-step HS atomizing method
HS2-W2@RF2			Two-steps HS atomizing method

### Material characterizations

The characterization of the samples was carried out in terms of color measurements (dyeing), water contact angle (finishing) and fastness of dyes and finishes towards washing and abrasion. As prepared dyed and finished wool fabrics were fully characterized to understand the effectiveness of the newly introduced advanced resource-efficient HS dyeing-finishing method. To study the dyeing performance, color measurements of the samples were done with a Datacolor 500 spectrophotometer. The color data was measured in the visible spectrum region of 360–700 nm and converted into tristimulus values that describe a specific point in the color space. With this measurement tool, two different color values were measured and used in the color assessment. The color strength was measured through Kubelka–Munk's equation (Eq. 1) using reflectance of dyed samples (R), the absorption coefficient (K) and the scattering coefficient (S), and expressed as K/S.

1 $$\frac{{\text{K}}}{{\text{S}}} = \frac{{\left( {1 - R} \right)^{2} }}{2R}$$

Besides the color strength, the color difference between samples was measured and expressed as the ΔE_CMC_ value. To get a mean value of the K/S and ΔE_CMC_ value, four different readings of reflectance on different positions on each sample were used. These measurements were done with three replicates of each sample. The samples were conditioned before measuring, and to get an accurate color measurement, the fabric was folded, so a double layer was measured. To assess the performance of the hydrophobic finish, hydrophobicity of the samples in terms of water contact angle was measured using an optical tensiometer from Biolin Scientific (Attension Theta). The water contact angle (${\theta }_{{\mathrm{H}}_{2}\mathrm{O}}$) was measured by taking the average contact angle after 2 s once the water droplet (drop size 5 μL) was stabilized on the fabric surface. Three independent measurements were carried out on each sample and the mean value with coefficient of variation has been reported. The fastness of dyes and finishes toward washing was measured according to ISO 6330:2012 in a wascator, type A. The samples were washed according to the program 4 N (as discussed in the standard), with a maximum load of 2 kg, at 40 ± 3 °C, for 30 min and with 20 ± 1 g of a non-phosphate powder detergent without optical brightener and enzymes. To test the color fastness over time, washing was repeated with another three cycles, with a drying step in between at 70 °C for 25 min and conditioned before further color testing. The fastness of dyes and finishes toward abrasion was measured using a Martindale SDL Atlas M235 with a speed of 47.5 revolutions per minute according to ISO 12,947–1:1998, a weight load of 9 kPa and a total of 10,000 runs. A standard woven wool fabric was used as the opposite rubbing cloth.

### Statistical analysis

Statistical analysis was performed to determine whether there is significant difference in the gathered data by implementing them in Minitab statistical tool. To determine the significant difference between processes (conventional, two-steps and one-step HS) a one-way ANOVA test was carried out at 95% confidence interval with the null hypothesis stating that all means are equal and the alternative hypothesis stating that at least one mean is different. To test the significant difference between the measurements before and after washing test, a paired t-test was performed at 95% confidence interval with the null hypothesis stating that the mean of differences (μ_d_) is equal to 0 and the alternative hypothesis stating that the mean of differences is not equal to 0.

## Results

The results from this study are presented in two parts; ***Part 1*** presents the results related to the dyeing performance of resultant wool fabrics prepared through two-steps or one-step HS method in relation to the conventional exhaust dyeing method. ***Part 2*** presents the results related to the finishing performance of resultant wool fabrics prepared through HS methods (two-steps or one-step) in relation to the conventional pad-dry-cure method.

### Part 1: Analysis of dyeing performance

A comparative study to assess the dyeing performance of the two-steps and one-step HS method in relation to the conventional exhaust dyeing method were carried out through color strength (K/S) measurements, color difference (ΔE_CMC_) measurements, and fastness to washing and abrasion based on color measurements of the dyes wool fabrics.

#### Color strength (K/S) measurement

**Figure 2:**
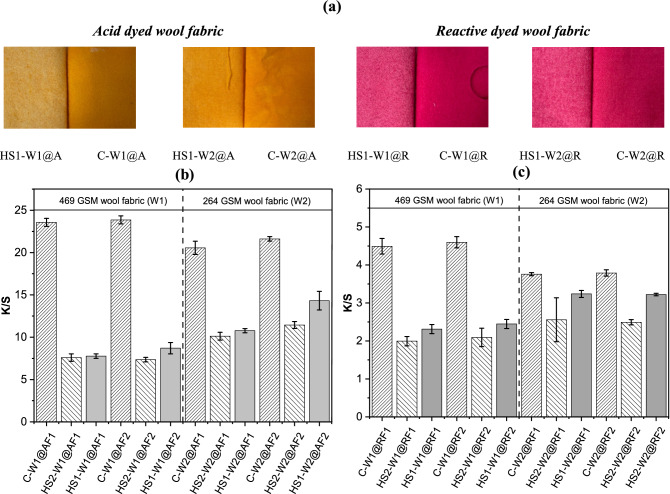
(**a**) Digital photographs of the samples; (**b**) Color strength (K/S) measurements of Acid dyed wool fabric; (**c**) Color strength (K/S) measurements of reactive dyed wool fabric ( = Conventional;   = Two-steps HS method;  = One-step HS method).

All dyed wool fabrics with both acid and reactive dyes were evaluated through color strength measurements to identify the difference between the conventional and HS dyeing method. In principle, the color strength value provides evidence related to the depth of the color on the dyed fabric surface^22^. Results presented in Fig. 2b and c show the plots of K/S values of the dyed wool fabric samples prepared with acid dye and reactive dye. Results from both acid and reactive dyed samples showed that there is a significant difference in color strength depending on the method of dyeing used, which is also visible by the naked eye (Fig. 2a). Conventionally dyed samples showed higher color strength values than HS dyed samples. This can be due to the possible diffusion limitation of dyes in the HS dyeing method compared to the conventional dyeing method, which restricted the dyes to be evenly distributed on the pores of the wool fabric^23^.

Higher diffusion in conventional methods may occur due to the use of electrolytes in the conventional method (which was not used in HS method) that influences the solubility and adsorption of dyes into the fibers^24^. The wool fiber swells in liquid, and the acidic conditions charge the amino acids on the surface, making it possible for the dye to enter the fiber and make strong bonds with the fibers^25^. As the dye fixation in the HS method is a rather dry process, where the fabric is subjected to only dry heat while being moist from the spray liquid, the wool fiber swells less, and affects the dye fixation process. The fabric surface keeps less moisture (the characteristic of wool) during the fixation, and the dyes migrate into a damper environment with lower pH. The level of unfixed dyes is not higher, so the fixation seems to take place deeper into the fabric. Nevertheless, the K/S value of samples dyed using new HS methods showed significant color strength as high as 14.0 which is suitable for commercial application.

A close look at the results shows that, there is a noticeable difference in color strength between the one-step and two-steps HS dyed-finished samples. One-step HS dyed samples showed better color strength than that of two-steps spray dyed samples. The poor color strength in the two-steps HS dyeing method can be due to meddling applied to the color during the finishing step. Although the dyeing method for both cases was the same, in the one-step method, dyes and finishes were mixed and sprayed over the wool fabric together, whereas in the two-steps method, dyes and finishes were sprayed separately over the samples to form a layer-by-layer assembly of dyes and finishes. The extend of the difference in color strength between the one-step and two-steps dyed samples were found to be influenced by the type of finishes used and the weight of the fabric (see Fig. 2). Finish 2 (Ruco-Dry DHE) was found to result in more color difference than Finish 1 (Ruco-Dry ECO DCF). Lighter weight wool fabric (W2/ 264 GSM) provided better color strength in HS dyed samples compared to heavier weight wool fabric (W1/469 GSM).

#### Color difference (ΔE_CMC_) measurement

**Figure 3:**
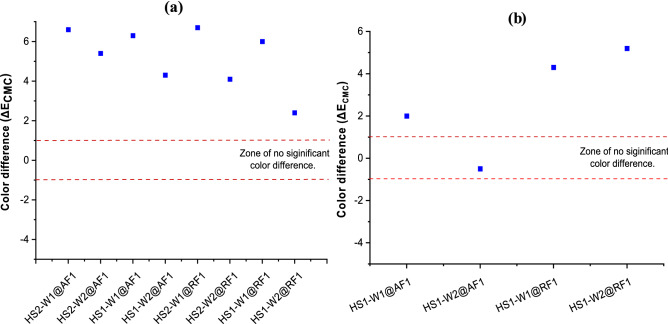
Color difference (ΔE_CMC_) between (**a**) HS dyed (two-steps) and conventional exhaust dyed samples; (**b**) One-step and two-steps HS dyed samples (shaded square = color difference points).

To further understand the dyeing performance of the conventional and HS method, the color difference of both acid and reactive dyed wool fabric samples were evaluated. At first, the comparative color difference analysis of the HS dyed samples (one-step) was carried out in respect to the samples prepared through conventional method (see Fig. 3a). After that, the color difference between one-step and two-steps HS dyed samples was studied, as well (Fig. 3b). Results from the color difference between HS dyed and conventional exhaust dyed wool fabric samples shows a significant color difference that can be detected by the naked eye as all samples showed a ΔE_CMC_ value of over 1.0. This indicates the possible color difference of the samples due to the dyeing condition in different combination of HS method (one-step or two-steps), wool fabric (W1 or W2), and finishing agent (F1 and F2) which can be subjected to optimization before bulk processes in industrial scale. Nevertheless, the color difference for W1 fabric (469 GSM) was found strongest with 6.6 for acid dyes and 6.7 for reactive dyes. For W2 fabric (264 GSM), the color differences are 4.5 for acid dyes and 4.1 for reactive dyes. This emphasizes the characteristics of two different textile processes to achieve altered product performances. A close look at the results reveals that acid dyes account for a higher color difference than reactive dyes. Reactive dyes are a better fit for a continuous dyeing process, as the dyeing mechanism is less dependent on the swelling of the wool fiber under high temperatures and presence of water^26,27^. On the other hand, the color difference in a one-step HS method is lower than two-steps method (see Fig. 3a). Further analysis on the color difference measurement between the two-steps and one-step dyed samples (see Fig. 3b) shows that W1 fabric dyed with either acid dyes or reactive dyes and finished with F1 has shown ΔE_CMC_ values, which are high enough to be detected by the naked human eye^28^. On the contrary, the color difference of W2 fabric dyed with acid dyes and finished with F1 has shown ΔE_CMC_ value less than 1, which indicates the existence of color difference beyond the detection limit of the human eye.

#### Fastness to washing based on color strength (K/S) measurement

**Figure 4:**
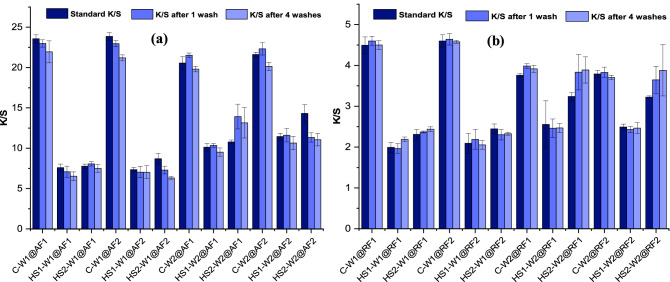
Fastness to washing based on color strength (K/S) measurement; (**a**) Acid dyed wool fabric; (**b**) Reactive dyed wool fabric ( = Standard K/S (left);   = K/S after 1 wash (middle);  = K/S after 4 washes (right)).

Color fastness is an essential analysis to determine the performance of dyeing. Resultant wool fabric samples prepared through either conventional or HS dyeing method were subjected to fastness to washing analysis. The dyeing performance over fastness to wash has been evaluated based on color strength measurements, which is plotted in Fig. 4. Results show that, regardless of the method used, K/S values of most samples have decreased after washing. The decrease in K/S after washing can be explained as the loss of loosely fixed dyes from the fabric during washing^29,30^. Some samples showed surprising increase in color strength after four washing cycles compared to one cycle, which can be due to the attainment of the evenness of dyes on the fabric surface after possible patches of dyes were removed. This novel study has opened several new discussions through its findings, where investigation of reported phenomenon of color strength related to washing is one of them. Although this is out of the scope of this work, but it certainly can be explored for better understanding of the HS technology for dyeing and finishing.

#### Fastness to washing based on color difference (ΔE_CMC_) measurement

Color fastness of resultant wool fabric in terms of washing has been further evaluated based on color difference of samples before and after washing. Results are presented in Table 2, which showed that the samples dyed with acid dyes are significantly different in color after washing (observed for both conventional and HS methods). Result shows that, W2 fabric holds its color better after washing than W1 fabric, as the ΔE_CMC_ values have a lower significant difference. A comparison between acid and reactive dyes shows that reactive dyes have a better washing fastness than acid dyes, as most ΔE_CMC_ values stay close to or < 1, which can be found for both types of fabric dyed with reactive dyes. In general, the color difference increased with the number of washes for samples prepared with all three methods (one-step HS method, two-steps HS method, and conventional method). A close look on the results provides evidence of comparatively higher color difference on the HS dyed samples then the conventionally dyed samples. The ΔE_CMC_ in HS1-W1@AF1 after one wash was 3.73 that rose to 5.34 after four washes, whereas C-W1@AF2 has an initial ΔE_CMC_ of 0.71 after one wash that rose to 1.50 after four washes. This can be due to the successive impact of the washing cycles on the interaction of the loosely attached/bonded dyes with the fabric surface that causes the abstraction of dyes from the fabric^31^. Nevertheless, despite the loss of dyes, the strength of color is high enough to retain the characteristics of the dyed fabric as a colored material as supported by the K/S analysis.

**Table 2:** Fastness to washing based on color difference (ΔE_CMC_) measurement.

Sample name	Color difference (ΔE_CMC_)	
	After 1 wash	After 4 washes
**Acid dyed wool fabric**		
C-W1@AF1	1.05	1.51
HS2-W1@AF1	3.73	5.34
HS1-W1@AF1	4.97	6.04
C-W1@AF2	0.71	1.50
HS2-W1@AF2	2.32	3.81
HS1-W1@AF2	1.58	3.51
C-W2@AF1	0.41	1.13
HS2-W2@AF1	2.06	3.95
HS1-W2@AF1	2.31	3.14
C-W2@AF2	0.70	0.86
HS2-W2@AF2	2.72	4.41
HS1-W2@AF2	3.52	4.74
**Reactive dyed wool fabric**		
C-W1@RF1	0.22	0.37
HS2-W1@RF1	0.94	1.27
HS1-W1@RF1	1.02	1.10
C-W1@RF2	0.32	0.45
HS2-W1@RF2	0.86	1.08
HS1-W1@RF2	1.49	1.33
C-W2@RF1	0.41	0.58
HS2-W2@RF1	0.98	0.92
HS1-W2@RF1	1.33	1.28
C-W2@RF2	0.30	0.34
HS2-W2@RF2	0.51	0.43
HS1-W2@RF2	0.97	1.36

#### Fastness to abrasion based on color strength (K/S) measurement

Color fastness of selected dyed wool fabrics in respect to abrasion was studied based on color strength K/S (before and after abrasion) according to the method described earlier (material characterizations section). Results shows that samples prepared through hydraulic spray atomizing system exhibited no significant difference in color strength after abrasion test regardless of one-step and two-steps process as well as dyes used. This phenomenon is particularly important as the hydraulic spray atomizing system is a continuous coloration process that excludes several after treatment process compared to conventional method. A detailed study can be carried out as a further study to understand the mechanism of superior color fastness of selected dyed wool fabric in respect to abrasion.

### Part 2. Analysis of finishing performance

To understand the effect of each preparation method and the performance of the hydrophobic finishes, all finished samples were comparatively studied through water contact angle measurement and their fastness in respect to washing and abrassion. A one-way ANOVA analysis was performed on the data to determine the significant difference between the samples.

#### Water contact angle (${\theta }_{{\mathrm{H}}_{2}\mathrm{O}})$ measurement

**Figure 5:**
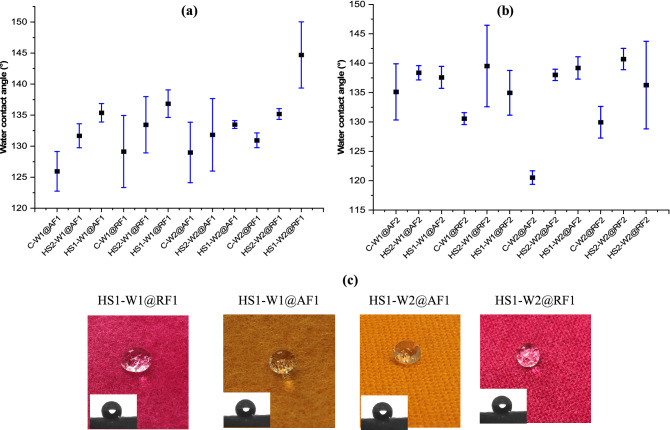
Water contact angle analysis of hydrophobic finished wool fabrics; (**a**) Ruco-Dry ECO DCF (F1); (**b**) Ruco-Dry DHE (F2) and (**c**) digital picture of hydrophobicity of wool fabric.

To assess the hydrophobicity of the samples, contact angle measurements were performed as described earlier in material characterizations section. Figure 5 shows the ${\theta }_{{\mathrm{H}}_{2}\mathrm{O}}$ of the wool fabric prepared with either Ruco-Dry ECO DCF (F1) or Ruco-Dry DHE (F2) finishes. Results show a significant difference in ${\theta }_{{\mathrm{H}}_{2}\mathrm{O}}$ of the finished wool fabric depending on the preparation method used. In general, all samples finished with any of the two finishes showed higher water contact angle on the fabric when it is prepared with the HS method compared to samples prepared through conventional padding method. This can be explained with the hydrophobic nature of wool which repels liquid to enter the core of the fiber or fabric^26,32^. As the finishing liquid most likely does not fully penetrate into the fabric, the water-repellent chemicals will primarily react with the fibers on the fabric surface, which results in higher contact angles for the HS finished samples compared to the conventional padded samples.

As for Ruco-Dry ECO DCF (F1), samples prepared with the conventional padding method for W1 fabric, a ${\theta }_{{\mathrm{H}}_{2}\mathrm{O}}$ of 125° was recorded, which can be increased by 9° if prepared through a two-steps HS method and by 14° if prepared through one-step HS method. This indicates a better finishing performance of the HS method over conventional padding method once a surface effect, in this case a water-repellency, is desired. Comparing the one-step and two-steps HS method shows that the one-step HS method is more efficient than the two-steps method. The high finishing performance is related to the even and uniform distribution of the finishes on the surface of the wool fabric. On the other hand, a comparison between W1 fabric and W2 fabric shows that there is no significant difference in finishing performance contrary to the difference in dyeing performance. For samples prepared with Ruco-Dry DHE (F2), samples prepared with the HS method show better finishing performance than conventionally padded samples. However, no significant difference was found between the one-step and the two-steps HS method. In general, the finishing performance of the samples prepared with Ruco-Dry DHE was found to be higher than that of Ruco-Dry ECO DCF. Samples finished with Ruco-Dry DHE approach superhydrophobic properties with contact angles between 140 and 150° for one-step HS samples^33,34^. Notable is also that there is no significant difference in average contact angles between the two different fabrics W1 and W2.

#### Fastness of hydrophobic finishes of wool fabrics to washing

The fastness properties of the applied finishes on the two wool fabrics have been studied in respect to washing as presented in Table 3. Results show that, in general, almost all samples decrease in the finishing performance after washing, which can be related to the removal of loosely attached or bonded finishes on the fabric surface. Comparing conventionally padded, one-step and two-steps HS finished samples, the loss of performance is more prominent in samples prepared with the one-step HS method followed by the two-steps HS method and lastly the conventional padding method. The differences in ${\theta }_{{\mathrm{H}}_{2}\mathrm{O}}$ for the samples prepared with HS methods can be related to the fact that the hydrophobic agents seem to make weaker bonds in a direct spraying process. As it is likely that there are more negatively than positively charged amino acids on the surface, the water repellent agents form weaker bonds with the fiber surface. During washing, these bonds are easily broken causing the fabric to lose some of its hydrophobicity^35^. Besides, there is no significant difference in average contact angles for all samples after washing, the samples of all three processes show similar contact angles. For W1 fabrics finished with Ruco-Dry Eco DCF these contact angles are 129° prepared by the conventional method and 132° and 130° in the two-steps and one-step HS method, respectively. This is similar for W2 fabrics with the same finish, where the contact angles respectively vary from 131° (conventional padding) to 133° (two-steps HS) and 132° (one-step HS). After a washing cycle, the samples were tumble dried to restore the full effect of the water-repellent finish. The realignment of the hydrophobic agent on the fiber surface can cause the contact angle to increase after washing, as seen with the conventional samples. In general, Ruco-Dry DHE performed worse in the washing test than Ruco-Dry Eco DCF, whereas the initial contact angles of DHE were higher than those of DCF as presented in Table 3.

**Table 3:** Fastness of hydrophobic finishes to washing based on water contact angle of the finished wool fabrics.

Sample name	${{\varvec{\theta}}}_{{{\varvec{H}}}_{2}{\varvec{O}}}$(°)	
	Before wash	After wash
**Ruco-Dry ECO DCF (F1)**		
C-W1@AF1	126 ± 3	127 ± 7
HS2-W1@AF1	132 ± 2	129 ± 1
HS1-W1@AF1	135 ± 1	131 ± 5
C-W1@RF1	129 ± 6	127 ± 3
HS2-W1@RF1	133 ± 5	136 ± 6
HS1-W1@RF1	137 ± 2	127 ± 7
C-W2@AF1	129 ± 5	135 ± 1
HS2-W2@AF1	132 ± 6	133 ± 2
HS1-W2@AF1	133 ± 1	134 ± 5
C-W2@RF1	131 ± 1	136 ± 4
HS2-W2@RF1	135 ± 1	125 ± 6
HS1-W2@RF1	145 ± 5	128 ± 3
**Ruco-Dry DHE (F2)**		
C-W1@AF2	135 ± 5	131 ± 2
HS2-W1@AF2	138 ± 1	135 ± 7
HS1-W1@AF2	138 ± 2	128 ± 2
C-W1@RF2	131 ± 1	127 ± 1
HS2-W1@RF2	140 ± 7	127 ± 4
HS1-W1@RF2	135 ± 4	131 ± 8
C-W2@AF2	121 ± 1	127 ± 1
HS2-W2@AF2	138 ± 1	132 ± 1
HS1-W2@AF2	139 ± 2	128 ± 1
C-W2@RF2	130 ± 3	130 ± 1
HS2-W2@RF2	141 ± 2	126 ± 4
HS1-W2@RF2	136 ± 7	132 ± 2

To further understand the differences among the samples prepared through all three methods, results were analyzed through a paired t-test. Table S1 of the supplementary information discusses the *P*-values of the performed paired t-test. If the *P*-value is below 0.05, the null hypothesis, i.e., the mean of the differences is 0, should be rejected. This means that the differences in means before and after washing are significantly different, when the *P*-value is lower than 0.05. A few samples show an insignificant difference in their contact angle before and after washing, although a consistency in the values is missing. Generally, the samples dyed and finished conventionally show a lower significance of difference.

#### Fastness of hydrophobic finishes of wool fabrics in respect to abrasion

Another factor that can affect the performance of finishes is abrasion. Therefore, the fastness of hydrophobic finishes on wool in respect to abrasion has also been studied based on the water contact angle measurement. Similar to fastness to washing, the performance of hydrophobic finishes was also affected by abrasion. In general, the loss of performance is more prominent in samples prepared with one-step HS followed by the two-steps HS and lastly by conventional padding as presented in Table 4. As was mentioned earlier, direct spraying in the HS method causes the hydrophobic finish to form less strong bonds because of the lack of positively charged amino acids on the fiber surface, which are thus more easily rubbed off.

**Table 4:** The t-values and *P*-values of the paired t-test performed on the contact angle measurements before and after abrasion.

Sample name	t-value	*P*-value	Mean ${{\varvec{\theta}}}_{{{\varvec{H}}}_{2}{\varvec{O}}}$ (°) before abrasion	Mean ${{\varvec{\theta}}}_{{{\varvec{H}}}_{2}{\varvec{O}}}$ (°) after abrasion
HS2-W1@F1	1.7	0.1128	134 ± 4	130 ± 5
HS1-W1@F1	6.5	< 0.0001	139 ± 6	129 ± 2
C-W1@F2	− 0.2	0.8394	131 ± 5	132 ± 5
HS2-W1@F2	3.0	0.0115	137 ± 3	132 ± 5
HS1-W1@F2	7.0	< 0.0001	140 ± 6	126 ± 4
HS2-W2@F1	2.2	0.0488	134 ± 3	130 ± 5
HS1-W2@F1	4.0	0.0019	139 ± 6	131 ± 5
C-W2@F2	− 1.2	0.2544	127 ± 4	129 ± 5
HS2-W2@F2	5.3	0.0002	139 ± 4	131 ± 7
HS1-W2@F2	11.6	< 0.0001	143 ± 4	128 ± 3

To determine whether the difference between the means before and after abrasion is significantly different, a paired t-test was carried out. Table 4 presents the t- and *P*-values that were gathered from the experiment as well as mean water contact angles before and after abrasion. If the *P*-value is below 0.05, the null hypothesis, i.e., the mean of the differences is 0, should be rejected. In most cases, this means that the contact angle of the samples dyed and finished conventionally are not significantly different before or after abrasion. All wool fabric samples dyed and finished in the HS methods, except for HS2-W1@F1, C-W1@F2, HS2-W2@F1 and C-W2@F2, however, show that there is no significant difference in the contact angle measurements before and after abrasion, as the *P*-values are below 0.05.

### Sustainable aspects of the HS atomizing process

The sustainable aspect of HS atomizing process has been investigated in terms of use of water, energy, and chemicals. Proposed HS methods are continuous dyeing-finishing process; thus, the processing time was not in the scope of the study. Nevertheless, the speed of the process is subjected to real-time optimization during bulk productions. Table 5 shows an overview of the resource consumption in different HS method compared to conventional methods. Results shows that, the HS methods attributes promising resource-efficient properties with saving of up to 88% water, 100% chemicals depending on the fabric and process it replaced. However, over 200% more dyestuff is used in this process, as the HS dyebath is much more concentrated which need to be optimized before large scale industrial applications. Along with records from Imago, it can be seen that, the new HS method reduces the consumption of energy, water and produces less waste that is identical to the sustainable trifecta -net zero energy, water and waste. Calculations on a large scale are very dependent on many variables, but a comparison of the lab scale methods can at least be made, which may allow predictions for industrial production. The contents of these baths can be translated to their contents per gram of fabric. When comparing the results between the one-step and two-steps HS method, it can be seen that the one-step method uses 50% less water because it combines two baths into one. Because of the shift in pick-up percentage from 60 to 80% of the water-repellent chemicals, 25% less of these chemicals were added to the one-step bath. Because of the higher content of the more alkaline wetting agent in the bath, more acetic acid needed to be added to adjust the pH value.

**Table 5:** Sustainable aspect of HS methods compared to conventional method.

Parameters	Two-steps HS-acid dye (%)	Two-steps HS-reactive dye (%)	One-step HS-acid dye and DCF finish (%)	One-step HS-acid dye and DHE finish (%)	One-step HS-reactive dye and DCF finish (%)	One-step HS-reactive dye and DHE finish (%)
Bath liquid	− 88	− 88	− 50	− 50	− 50	− 50
Water repellent	–	–	− 25	− 25	− 25	− 25
Dyestuff	213	213	–	–	–	–
Acetic acid	− 88	− 88	186	173	0	− 33
Sodium sulphate	− 100	− 100	–	–	–	–
Wetting agent	− 88	− 88	88	88	88	88

The reactive dyebath is less acidic and thus requires less acidic acid to balance the pH. The reduction in energy used comes from the dye fixation process. In an exhaust dyeing process, the bath liquid has to stay at a certain temperature throughout the process, which is energy consuming. The different method for dye fixation, in an autoclave as opposed to a heated and moving bath, cause the reduction in energy use. The wastewater generated in the HS method is also less than in a conventional process. The dye or finish liquid is used almost entirely, reducing the wastewater from the dyeing, and finishing process.

## Conclusions

In summary, this work introduces a new method for dyeing and finishing of wool fabric using hydraulic spray atomising process. The new method found successful in dyeing of wool fabric with both reactive and acid dyes in an ambient condition. The finishing of hydrophobic agent on dyed wool fabric was also achieved with great performance. The performance of dyeing and finishing as observed through color strength (K/S), color difference (ΔE_CMC_), color fastness analysis and (${\theta }_{{\mathrm{H}}_{2}\mathrm{O}}$) analysis establoshed the feasibility of the new methods as summarized below;

- Resultant wool fabric showed significant color strength as high as 14 which offers possibility to dye different depth of color ranging from medium to dark shades. When compared between one-step and two-steps HS methods, it appears that one-step HS method results in higher color strength and lower color difference while offering fastest and eco-friendly route for wool dyeing.
- The hydrophobic finish in wool fabric through HS method offered better performance than the conventional padding method. While HS method achieve ${\theta }_{{\mathrm{H}}_{2}\mathrm{O}}$ as high as 145° which is close to the super-hydrophobicity, the highest ${\theta }_{{\mathrm{H}}_{2}\mathrm{O}}$ through padding method was 135°.
- The HS method is indeed a water, energy, and chemical efficient method, with an 88% reduction in use of water compared to the conventional dyeing-finishing method. Less auxiliaries were used during the dyeing process because of the accuracy of the HS machine. The HS method positively affects the trifecta of sustainable development by improving the environmental, social and economic aspects.

The results reported in this study are of great importance as it established HS atomizing process as novel resource-efficient textile process towards a scalable technique. The results based on the lab-scale process predict promising conditions for a true-scale industrial process, where crucial reductions in the use of water and chemicals and hence less polluted wastewater are some of the main advantages. The overall results also emphasizes that the environmental benefits need to be weighed against the performance of the dyed and finished fabrics with the such novel processes.

## Supplementary Information

Supplementary Information.

## Data Availability

All data generated or analyzed during this study are included in this published article [and its supplementary information files].
