# Supplementary material for: Study on hydraulic spray atomizing system as a new resource-efficient dyeing-finishing method for wool fabric
Source: Sci Rep. 2022 Dec 17;12:21814. doi: 10.1038/s41598-022-26172-4 (PMC9759535; doi:10.1038/s41598-022-26172-4)
Supplement: Supplementary file 1 — Supplementary Information. [file 41598_2022_26172_MOESM1_ESM.docx]

**-----------------Supporting information----------------**

**Study on hydraulic spray atomizing system as a new resource efficient dyeing-finishing method for wool fabric**

Roos Mulder^1^, Mohammad Neaz Morshed^1, *^, Sina Seipel^1, *^, Ulrika Norén^1^, Ellinor Niit^2^, Vincent Nierstrasz^1^

^1^ Textile Material Technology, Department of Textile Technology, Faculty of Textile, Engineering and Business, University of Borås, Borås, Sweden

^2^ Imogo AB, 216 16 Limhamn, Sweden

*Corresponding authors email address: [mohammad_neaz.morshed@hb.se](mailto:mohammad_neaz.morshed@hb.se) (Mohammad Neaz Morshed), [sina.seipel@hb.se](mailto:sina.seipel@hb.se) (Sina Seipel)

1. **EXPERIMENTAL**
   1. ***MiniMax hydraulic spray atomizing system from Imogo AB***

Mini-Max is a standalone laboratory unit that is designed for sample dyeing and finishing. The device as shown in Figure S1 has two spraying nozzles that move from left to right to spray the surface. The spraying chamber is closed with a lid, and in the middle is a sample bed to rest the sample on. The container with the dyeing or finishing bath in it is attached to the hydraulic spray atomising system in the back. Pressure is also added here, and a pressure of 6 bar was used to prepare the samples. A compensation factor was used when necessary, in order to compensate the output of the nozzles to get the correct pick-up percentage. A different compensation factor was used for almost every bath, depending on how much had to be compensated. The MiniMax sprays only one side of the fabric, which also provides the option to only add color or functionalization on one side of the fabric. In this experiment, the samples were however sprayed and finished on both sides. The samples were dry when sprayed, but conditioned beforehand. The bath temperature was around 20°C.


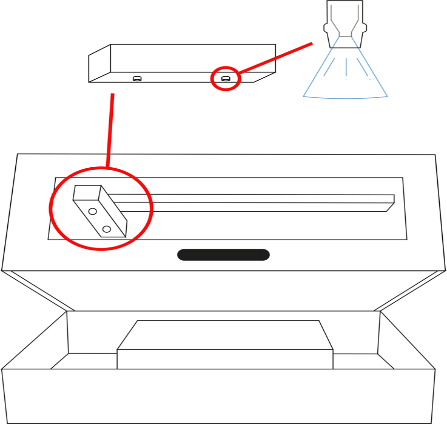

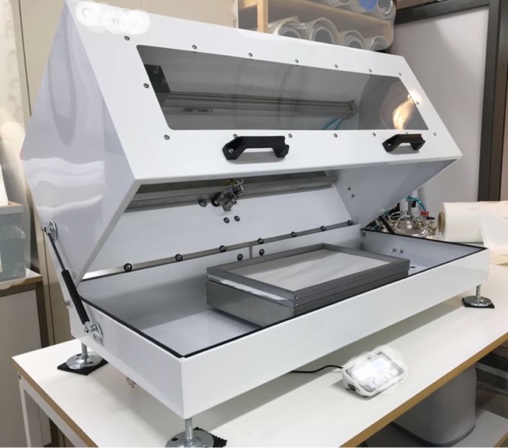


**(a)**

**(b)**

**Figure S1:** (a) Schematic illustration and (b) Picture of MiniMax hydraulic spray atomizing system.

- 1. ***Fastness of hydrophobic finishes of wool fabrics to washin*g**

**Table S1**: The t-values and P-values of the paired t-test performed on the contact angle measurements before and after washing.

| Sample name | t-value | P-value | Mean before washing [°] | Mean after washing [°] |
| --- | --- | --- | --- | --- |
| HS2-W1@F1 | 0.8 | 0.4333 | 134 ± 4 | 132 ± 6 |
| HS1-W1@F1 | 4.3 | 0.0013 | 139 ± 6 | 130 ± 5 |
| C-W1@F2 | -0.2 | 0.8615 | 131 ± 5 | 132 ± 3 |
| HS2-W1@F2 | 3.7 | 0.0033 | 137 ± 3 | 131 ± 4 |
| HS1-W1@F2 | 5.5 | 0.0002 | 140 ± 6 | 127 ± 5 |
| HS2-W2@F1 | 0.2 | 0.8140 | 134 ± 3 | 133 ± 6 |
| HS1-W2@F1 | 3.8 | 0.0031 | 139 ± 6 | 132 ± 4 |
| C-W2@F2 | -1.2 | 0.2638 | 127 ± 4 | 128 ± 2 |
| HS2-W2@F2 | 4.4 | 0.0010 | 139 ± 4 | 131 ± 4 |
| HS1-W2@F2 | 8.6 | < 0.0001 | 143 ± 4 | 130 ± 3 |
